# SARS-CoV-2 Helicase (NSP13) Interacts with Mammalian Polyamine and HSP Partners in Promoting Viral Replication

**DOI:** 10.3390/cimb48010080

**Published:** 2026-01-13

**Authors:** Zingisa Sitobo, Liberty T. Navhaya, Ntombekhaya Nqumla, Madipoane Masenya, Matsheliso Molapo, Yamkela Mthembu, Sesethu Godlo, Xolani H. Makhoba

**Affiliations:** 1 Department of Biochemistry and Microbiology, University of Fort Hare, Alice Campus, Alice 5700, South Africa; 2 Department of Biochemistry, Microbiology and Biotechnology, University of Limpopo, Turfloop Campus, Sovenga 0727, South Africa; 3 Department of Life and Consumer Sciences, College of Agriculture and Environmental Sciences, University of South Africa (UNISA), Florida Campus, Roodepoort 1709, South Africa; 4 Human Genetics Department, Computational Biology Division, Faculty of Health Sciences, University of Cape Town, Barnard Fuller Building, Anzio Rd, Observatory, Cape Town 7935, South Africa

**Keywords:** SARS-CoV-2, COVID-19, NSP13, ODC, polyamines, heat shock proteins, MD stimulations

## Abstract

We present a computational study that precedes the potential interactions between SARS-CoV-2 helicase (NSP13) and selected host proteins implicated in chaperone-assisted folding and polyamine metabolism. Using structure-based modelling and protein–protein docking (BioLuminate v4.6), followed by all-atom molecular dynamics (MD) simulations (GROMACS v2018.6), and comparative MM-GBSA scoring (HawkDock v2), we evaluated the stability and interface properties of NSP13 complexes with cytosolic heat shock proteins; heat shock protein 40 (HSP40), heat shock protein 70 (HSP70), heat shock protein 90 (HSP90) and the polyamine biosynthesis enzyme ornithine decarboxylase (ODC). Docking, MD, and interface analyses indicate distinct complex behaviours: HSP70-NSP13 complexes sampled compact conformations, HSP90-NSP13 ensembles displayed greater conformational heterogeneity but more favourable comparative MM-GBSA estimates, and ODC-NSP13 interfaces were comparatively well packed. Per-residue contact mapping identified a small set of recurrent NSP13 residues, Lys22 and Asn51, as putative interaction hotspots. The reported findings herein generate testable hypotheses about NSP13 recruitment of host chaperones and modulation of polyamine metabolism that may inform downstream experimental studies.

## 1. Introduction

The COVID-19 pandemic has impacted over a million individuals globally, resulting in thousands of fatalities, as an unforeseen health crisis [1]. Vaccines were developed to treat or prevent COVID-19, thus decreasing the mortality rate. Subsequently, a renewed sense of hope emerged for people, enabling them to reclaim their freedom, thus signifying a restoration of normalcy [2]. COVID-19 is caused by severe acute respiratory syndrome coronavirus 2 (SARS-CoV-2), which belongs to the *Coronaviridae* family. The transmission of SARS-CoV-2 has been reported to occur through respiratory droplets released during coughing, sneezing, as well as contact with contaminated surfaces, followed by contact with the mucous membrane, such as that of the mouth or of the nose [3]. SARS-CoV-2 consists of different types of helicases, which are known for their vital role in unwinding nucleic acids [4]. SARS-CoV-2 non-structural protein 13 (NSP13) is one of the SARS-CoV-2 helicases that use the energy obtained from nucleotide triphosphate hydrolysis to unwind the double-stranded nucleic acids. This particular helicase has been targeted as a potential biomarker because of its high conservation and critical role in viral replication.

NSP13 has a molecular weight of 67 kDa and is classified as a Helicase Superfamily 1B enzyme. It is responsible for the hydrolysis of nucleotides to initiate the unwinding process of DNA or RNA in a 5′ to 3′ direction [5,6]. Although it is thought to interact with RNA in vivo, in vitro enzyme characterisation indicates more robust DNA activity with relatively weak non-processive helicase activity when compared to other helicase enzymes in the superfamily [7]. NSP13 interacts with other non-structural proteins such as Nsp12, an RNA-dependent RNA polymerase, NSP7, NSP8, and NSP12 replication–transcription complexes. These interactions stimulate NSP13 helicase activity, possibly through mechano-regulation [8,9].

Ornithine decarboxylase (ODC) is known to initiate the polyamine production pathway. ODC is highly controlled, and its levels vary in response to several growth factors, oncogenes, and tumour promoters, as well as changes in polyamine concentrations. Its activity also varies in response to various inputs [10,11]. Variations in the amount of ODC protein turnover induce fluctuations in activity. A protein called antizyme controls ODC turnover by reacting to polyamine levels. ODC is similarly subject to transcriptional regulation. The Myc/Max transcription factor targets several genes, including the ODC gene. ODC mRNA is regulated at a third level during transcription [12,13].

Molecular chaperones are proteins known for their vital housekeeping functionalities in the cell [14]. Heat shock protein 40 (HSP40), known as DnaJ in prokaryotes, is a multi-member co-chaperone family. It is identified by a conserved J domain in its structure, which has approximately 70 residues and is used to induce heat shock protein 70 (HSP70) [15]. HSP40 is a co-chaperone that coordinates the activation of the HSP70 ATPase with substrate delivery to HSP70, thus facilitating protein folding, transport, and degradation functions [16]. HSP70 is an integral part of the cellular network of molecular chaperones and folding catalysts [17]. Heat shock protein 90 (HSP90) is the primary chaperone that assists in proper protein folding and stabilisation [18]. Historically, HSP90 was first identified in complexes with steroid hormone receptors and oncoprotein viral kinases. Since then, hundreds of HSP90 clients, essential in cellular processes often associated with cell growth and proliferation ([19] have been identified).

SARS-CoV-2, like any other pathogen, is dependent on host biomolecules for survival. When it enters the human body, it hijacks host proteins and polyamines that assist in viral entry, replication, and proliferation. The previously approved vaccines have been rendered ineffective toward COVID-19 due to the fast mutation rate of the SARS-CoV-2 spike glycoprotein, resulting in many emerging variants of concern [20]. There are still reports of newly recorded COVID-19 cases, which underscore the need for continuous research to identify potential biomarkers and effective antivirals toward the development of COVID-19 treatment. To our knowledge, no currently available study has reported the interaction of heat shock proteins, specifically HSP40, HSP70, and HSP90, with NSP13 and the interaction of ODC and the viral NSP13. Hence, this study aimed to elucidate the novel interactions between polyamines and heat shock proteins in the functional processes of these proteins.

## 2. Materials and Methods

### 2.1. Sequence Analysis of Selected Heat Shock Proteins, Polyamine, and nsp13

Sequences for the host heat shock proteins (HSP40, HSP70, and HSP90), ornithine decarboxylase (ODC) enzyme, and SARS-CoV-2 helicase (NSP13) were obtained from the NCBI database (https://www.ncbi.nlm.nih.gov) [21]: SARS-CoV-2 NSP13 (accession number: YP_459942.1), human heat shock proteins, heat shock protein 40 (accession number: P25685), heat shock protein 70 (accession number: NP_002145.3), and heat shock protein 90 (accession number: NP_001017963.2), and ornithine decarboxylase (accession number: P11926). Sequence analysis was performed using the NCBI database focusing primarily on the protein’s chromosome location, assembly, locus, gene ID, exons, and introns [22].

### 2.2. Homology Modelling

Homology modelling was performed using BioLuminate v4.6 (Schrödinger Release 2023-2), using the one-target/one-template strategy [23]. The identified experimental structures of the proteins of interest had missing amino acid residues; consequently, homology modelling was used to fill in the missing amino acid residues. The target sequences (FASTA) were searched against the PDB via the BLAST-based ‘Find’ tool under ‘Specify a template structure’. Template selection (identified available experimental structures) prioritised high sequence identity, alignment coverage, and BLAST alignment statistics (E-value). Pairwise alignments between target and template were inspected manually. All templates had ≥75% sequence identity. For the viral NSP13 protein, the 3D structure with PDB ID 6SZL (chain A; resolution of 1.94 Å; solved with X-ray diffraction) was used as the template for homology modelling. For the host HSP40 protein, the experimental structure 3AGZ was used; for the HSP90 protein, the template structure 7KRJ was used; and for the ornithine decarboxylase enzyme, the structure 2OO0 was used as the homology template. The candidate structures were modelled for each target, and their loops were refined and subjected to energy minimisation using the OPLS4 force field. The quality of the modelled structures was validated using a web-based tool, PDBsum (https://www.ebi.ac.uk/thornton-srv/databases/pdbsum/Generate.html (accessed on 15 June 2025)) [24].

### 2.3. Molecular Docking

Protein preparation of the candidate proteins before molecular docking simulations was performed following a method similar to that reported by Navhaya et al. (2024) [20]. All proteins were prepared using the ‘Protein Preparation Workflow’ of BioLuminate v4.6. Missing loops were added using ‘Prime’, hydrogens were replaced, and bond orders were assigned under the ‘Preprocess’ step of the ‘Protein Preparation Workflow’. Hydrogen bond assignment was optimised using ‘PROPKA’. Energy minimisation of the proteins was performed using the OPLS4 force field. Protein–protein docking was performed using the ‘Protein-Protein Docking’ tool under Biologics, with the host proteins (HSP40, HSP70, HSP90, and ODC) being regarded as the receptor, whilst the viral NSP13 protein was regarded as the ligand. The protein–protein interactions were analysed using the Protein Interaction Analysis tool of BioLuminate v4.6 [25].

### 2.4. MD Simulations

Molecular dynamics (MD) simulations were performed using GROMACS (v2018.6) [26]. For the docked complexes (HSP40-NSP13, HSP70-NSP13, HSP90-NSP13, and ODC-NSP13), the AMBER99SB-ILDN force field was assigned to the systems. A triclinic simulation box was placed on all protein complexes with a buffer size of 1.8 nm. The systems were solvated using the SPC/E water model. The system was neutralised using *gmx grompp* and *gmx genion* commands, adding Na^+^/Cl^−^ [26,27]. Energy minimisation was performed with relaxed protein complex systems using the steepest descent algorithm, which applied a force tolerance of 1000 kJ/mol/nm, with an upper limit of 50,000 steps. The computational resources from the Centre for High-Performance Computing (CHPC) cluster were utilised in system equilibration regarding pressure and temperature. Temperature equilibration was performed using a constant particle number, volume, and temperature ensemble, at 300 K, using the velocity rescale (v-scale) thermostat at a time step of 2 fs. Pressure equilibration was performed at 1 atm using the NPT ensemble with a maximum of 50,000 steps at a time step of 2 fs, using a Berendsen barostat. Both equilibrations were run for 100 ps. For each complex, the production of MD was performed at the CHPC using *gmx mdrun* [26,27], with HSP40-NSP13 complex simulation being run for 130 ns, HSP70-NSP13 complex simulation being run for 150 ns, HSP90-NSP13 complex simulation being run for 180 ns, and the ODC-NSP13 complex simulation being run for 200 ns. The LINCS method was used to restrain the bond lengths. Long-range electrostatics were handled using the Particle Mesh Ewald (PME) method, with short-range nonbonded interactions treated with cutoff values of 1.0. Production pressure coupling used the Parrinhelo–Rahman barostat, while temperature coupling used the V-rescale thermostat. For each complex, three independent replicates were performed to elucidate the reproducibility of the simulations.

### 2.5. Post-MD Simulations

Post-MD analyses were performed on the HSP40-NSP13, HSP70-NSP13, HSP90-NSP13, and ODC-NSP13 complexes using the simulation trajectories and the GROMACS topology files (xtc, tpr, and pdb). Structural metrics, including root mean square deviation (RMSD), root mean square fluctuation (RMSF), and radius of gyration (Rg), were computed using GROMACS tools (*gmx rms*, *gmx rmsf*, and *gmx gyrate*) using the alpha-carbon (Cα) atoms for RMSD and RMSF, and residues for radius of gyration [26].

Conformational clustering of the molecular dynamics’ trajectory was performed to identify dominant conformational states sampled during the MD production run. Clustering analysis was carried out with the Cα atoms using the GROMACS (*gmx cluster*) tool using the GROMOS algorithm and a 0.20 nm cutoff. The last 30 ns of the trajectories from each complex was used for clustering to focus on the equilibrated portion of the simulation at a time step of 200 picoseconds (ps) [26].

Hydrogen bond calculation was performed to analyse the interactions between the host and viral proteins from the HSP40-NSP13, HSP70-NSP13, HSP90-NSP13, and ODC-NSP13 complex systems with time. The calculation was performed using the GROMACS tool *gmx hbond*, with a cutoff distance of 0.35 nm and an angle of 30° on the previously mentioned trajectory timeframes for each complex system [26].

The MM-GBSA method was used in HawkDock Server (v2) (http://cadd.zju.edu.cn/hawkdock/; accessed on 2 August 2025) to compute binding free energies (ΔG_bind_). The results of the MM-GBSA, the pre-processed PDB files of the receptor and ligand, were downloaded and analysed [28].

## 3. Results

### 3.1. Sequence Analysis of Selected Heat Shock Proteins, ODC Enzyme, and NSP13

The field of sequence analysis is broad and encompasses a wide range of subdomain studies. Aligning sequences can provide essential information regarding a sequence’s functional and structural sites. It is employed to investigate the evolution of sequences by determining the sequence orthologues and homologues. Sequence analysis of proteins aims to determine the potential amino acid residues necessary for the desired alteration. In this study, sequences of the host proteins were retrieved from NCBI databases using their accession numbers. Sequence analysis was performed by looking into the genomic locus, chromosome number, number of introns, and number of exons (see Table S1).

Sequence analysis revealed that the proteins are located on different chromosomes. HSP40 is located on chromosome 19, HSP70 on chromosome 5, HSP90 on chromosome 14, and ornithine decarboxylase located on chromosome 2. HSP90 and ornithine decarboxylase have the same number of exons (13) and introns (12), while HSP40 has 8 exons and 7 introns, and HSP70 has 19 exons and 18 introns, as shown in Table S1. When genes are transcribed, the exons and introns are incorporated into the messenger RNA products, but they do not affect how these proteins function or interact with the cells continuously. However, during the splicing process, introns are removed, leaving only exons in the final mRNA, which are used to choose which proteins are produced.

Homology (comparative) modelling is a template-based computational method to predict a protein’s three-dimensional (3D) structure. In the presence of high-identity, high-resolution templates, homology models can be accurate. However, template-free (de novo) and modern deep learning methods, such as AlphaFold2 and RoseTTAFold, have substantially advanced the field and can outperform traditional homology modelling for targets lacking close templates [29]. In this study, BioLuminate v4.6 was used to predict the three-dimensional structures of heat shock proteins (HSP40, HSP70, and HSP90), the ODC enzyme (ornithine decarboxylase), and SARS-CoV-2 helicase (NSP13). These models were generated by aligning the sequences of an identified experimental template structure, duplicating the coordinates, and rearranging residues based on the sequence. Table 1 shows the identified template structures for all the proteins, including the parameters used to ensure that the template structures closely resemble the compositions of the amino acid residues and the overall architecture. Templates with the highest sequence identity were selected, and high sequence alignment was selected. Low E-values provided additional confidence in the reliability of the alignments. HSP70 lacked a fully identical template; therefore, we used a template structure (PDB ID 6GFA) with 75% identity, which we acknowledge might affect the functional and interface predictions particularly.

**Figure 1:**
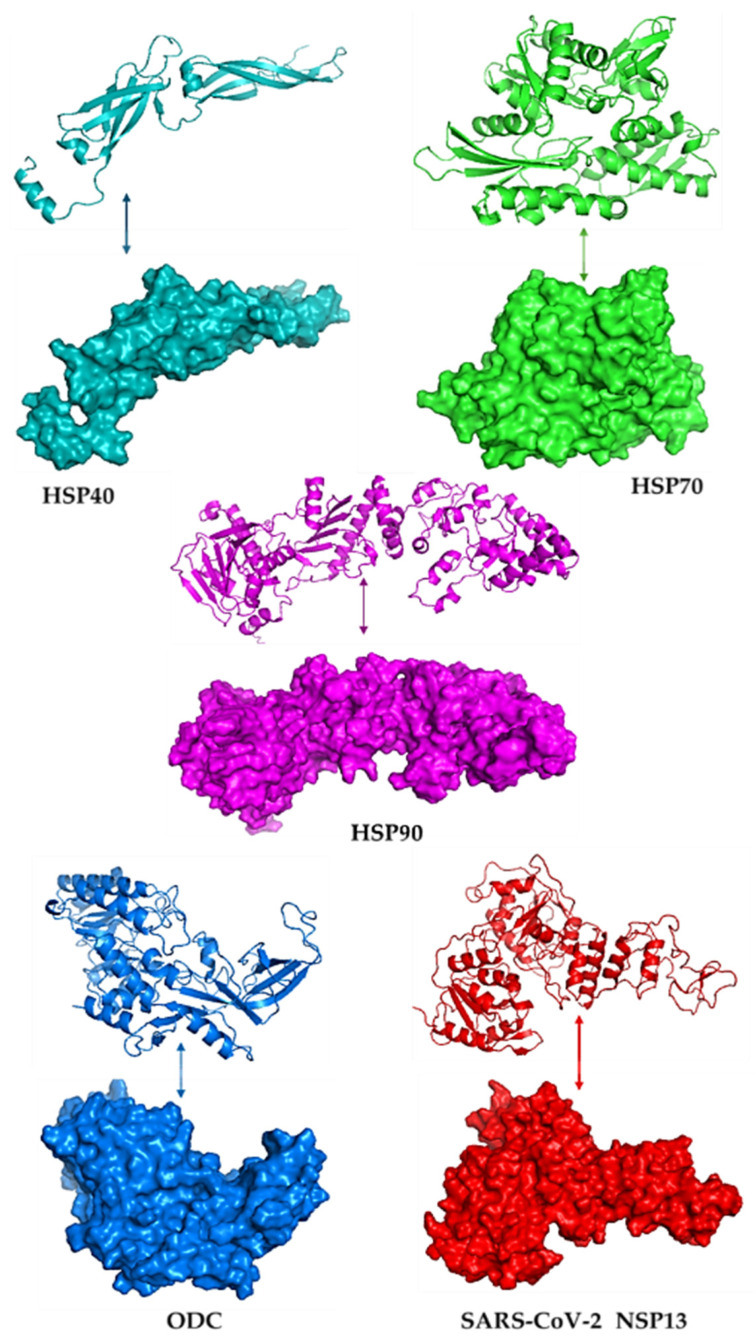
The 3D models and surface structures for the human heat shock proteins (HSP40, HSP70, and HSP90), polyamine-regulating enzyme called ornithine decarboxylase (ODC), and SARS-CoV-2 helicase (NSP13), visualised using PyMOL v3.1.6.1.

Figure 1 illustrates the modelled three-dimensional structures of the selected proteins. The quality of the modelled structure was validated using PDBsum, and the output was given as Ramachandran plots. In the core region of Ramachandran plots, PROCHECK states that for a high-quality model with outstanding stereochemical characteristics, the percentage of residues in the most favoured areas should be 90%. Figure S1 shows the Ramachandran plots of the modelled proteins, highlighting residues in the most preferred, permitted, liberally allowed, and banned regions. These structures were also subsequently tested using ERRAT, and the results showed that they were high-quality models with some scores over the cutoff (see Supplementary Figure S2). The total quality element was noted as their percentage of results. The problematic or unfavourable portions of the structure are represented by the colours red and yellow, whereas the typical parts are defined by the colour white.

### 3.2. Molecular Docking of NSP13 with Heat Shock Proteins and ODC Enzyme

**Figure 2:**
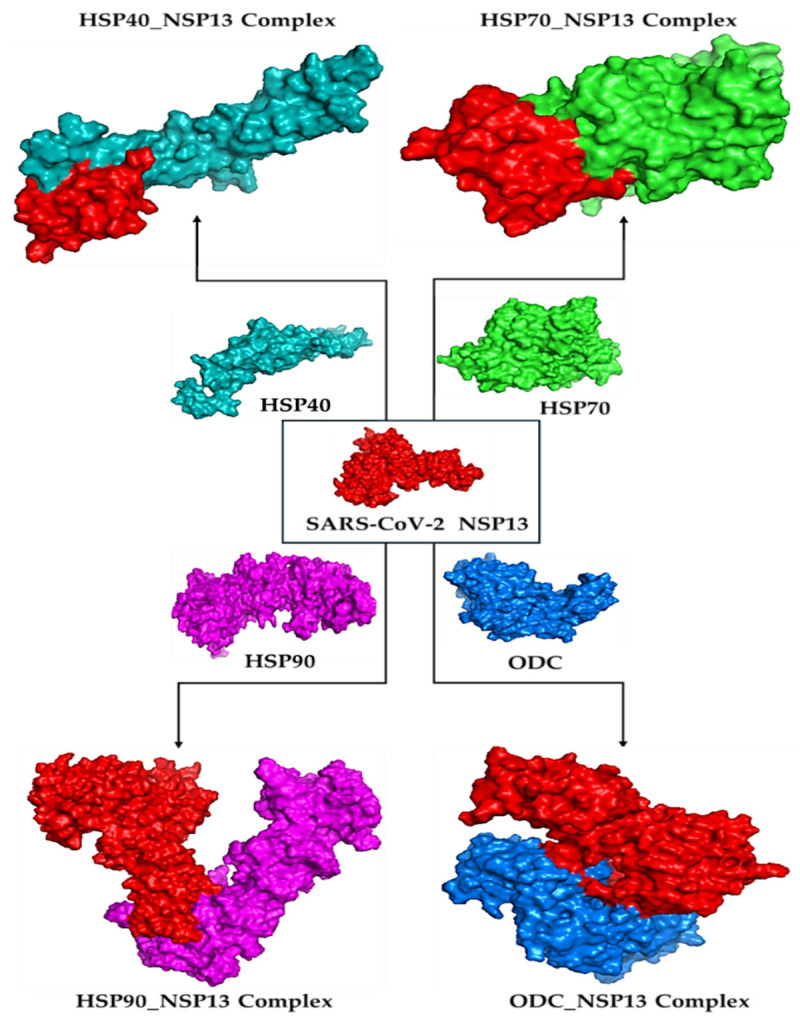
Docked complexes for HSP40, HSP70, HSP90, and ODC enzyme binding with NSP13 as visualised using Discovery Studio v21.1.0.20298.

**Figure 3:**
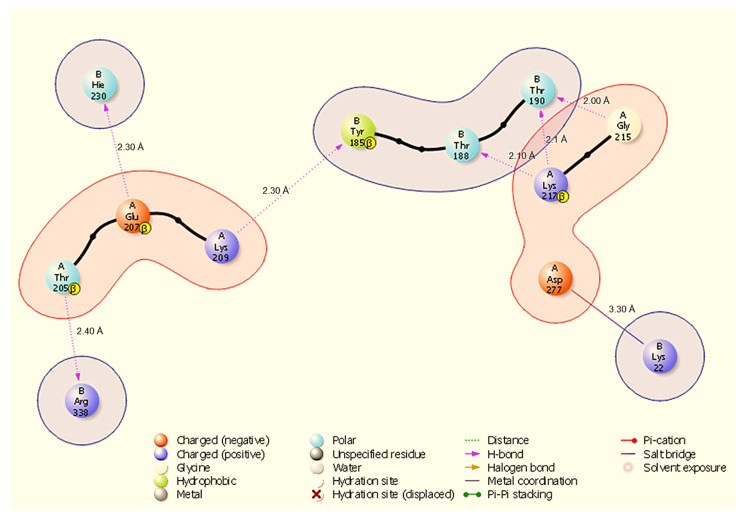
Two-dimensional interaction diagram showing hydrogen bonds and a salt bridge at the HSP40-NSP13 interface and their respective bond distances. Key annotated contacts: A:Gly215-B:Thr190 (H-bond, 2.00 Å), A:Lys217-B:Thr190 (H-bond, 2.10 Å), and A:Asp277-B:Lys22 (salt bridge, 3.30 Å).

**Figure 4:**
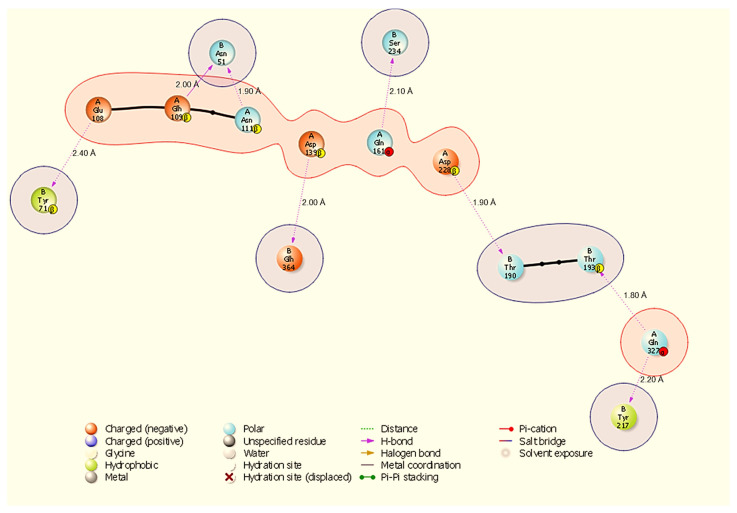
Two-dimensional diagram highlighting a binding hotspot in the HSP70-NSP13 complex, centred on A:Gln327 and multiple contacts to NSP13 B:Asn51 and B:Tyr217. Representative contacts: A:Gln327-B:Thr193 (H-bond, 1.80 Å) and A:Asp228-B:Thr190 (H-bond, 1.90 Å).

**Figure 5:**
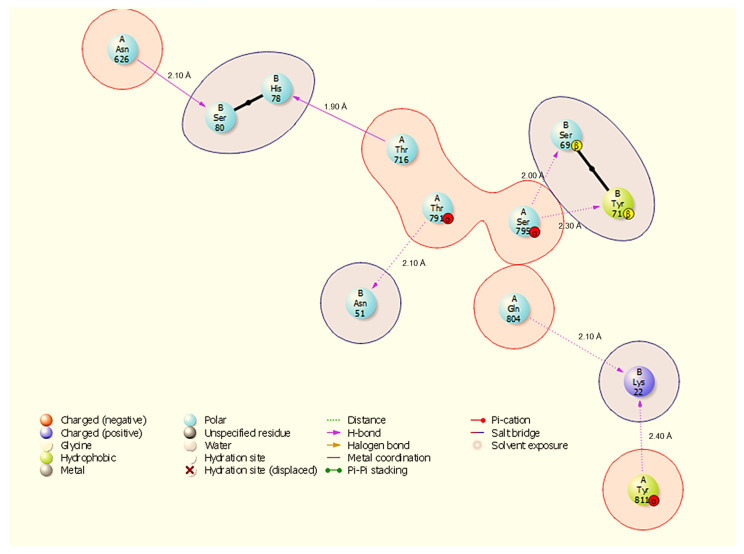
Two-dimensional interaction diagram showing hydrogen bonds and a salt bridge at the HSP90-NSP13 interface and their respective bond distances. Representative contacts: A:Thr716-B:His78 (H-bond, 1.90 Å) and A:Gln804-B:Lys22 (H-bond, 2.10 Å).

**Figure 6:**
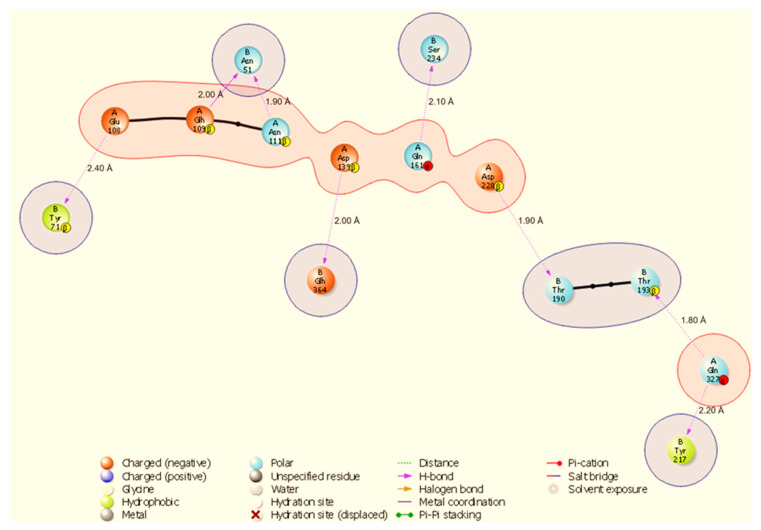
Two-dimensional interaction diagram showing dense hydrogen bonding in the ODC-NSP13 complex, involving A:Asn3 and a salt bridge A:Glu106-B:Arg21 (3.30 Å). Representative contacts: A:Asn3-B:Glu168 (H-bond, 2.10 Å) and A:Asn3-B:Lys171 (H-bond, 2.20 Å).

Molecular docking is a way to model how a small molecule interacts with a protein at an atomic level. This way, we can determine how small molecules interact with proteins at the binding site and understand fundamental biological processes. Figure 2 shows the docked complexes and poses of the host proteins with the viral protein from BioLuminate visualised using Discovery Studio. Protein–protein interaction analysis between SARS-CoV-2 NSP13 (chain B) and four host proteins (chain A: HSP40, HSP70, HSP90, and ODC) was performed using BioLuminate v4.6 and visualised as two-dimensional interaction diagrams (Figure 3, Figure 4, Figure 5 and Figure 6).

The docked interfaces are dominated by directional hydrogen bonds with only two salt bridges observed across the four complexes; hydrogen bond distances measured in the BioLuminate output range from 1.80 Å to 2.40 Å, while identified salt bridges measure 3.30 Å. Overall, the interfaces show chaperone-specific binding modes: HSP70 and HSP90 engage NSP13 primarily through short, highly directional hydrogen bonds (many ≤ 2.10 Å), HSP40 uses multiple hydrogen bonds plus one stabilising salt bridge, and ODC forms the largest number of hydrogen bonds but also one salt bridge that likely contributes to electrostatic anchoring.

In the HSP40-NSP13 complex (Figure 3), six discrete hydrogen bonds (H-bonds) were identified: A:Gly215-B:Thr190 (2.00 Å), A:Lys217-B:Thr190 (2.10 Å), A:Lys217-B:Thr188 (2.10 Å), A:Lys209-B:Tyr185 (2.30 Å), A:Glu207-B:Hie230 (2.30 Å), and A:Thr205-B:Arg338 (2.40 Å). In addition, A:Asp277 forms a salt bridge with B:Lys22 (3.30 Å). These contacts create a mixed polar interface in which A:Lys217 contributes multiple hydrogen bonds to closely spaced NSP13 threonine residues (B:Thr190, B:Thr188), and A:Asp277 provides a discrete electrostatic anchor to B:Lys22.

The HSP70-NSP13 interface (Figure 4) is characterised by eight hydrogen bonds comprising A:Gln327-B:Thr193 (1.80 Å), A:Asp228-B:Thr190 (1.90 Å), A:Asn111-B:Asn51 (1.90 Å), A:Asp139-B:Glh364 (2.00 Å), A:Glh109-B:Asn51 (2.00 Å), A:Gln161-B:Ser234 (2.10 Å), A:Gln327-B:Tyr217 (2.20 Å), and A:Glu108-B:Tyr71 (2.40 Å). Notably, A:Gln327 engages NSP13 at two distinct sites (B:Thr193 and B:Tyr217), indicating a localised HSP70 hotspot; NSP13 B:Asn51 is contacted by multiple host residues (A:Asn111 and A:Glh109), identifying B:Asn51 as a recurring contact residue on NSP13.

HSP90 binds NSP13 through seven hydrogen bonds (Figure 5): A:Thr716-B:His78 (1.90 Å), A:Ser795-B:Ser69 (2.00 Å), A:Gln804-B:Lys22 (2.10 Å), A:Thr791-N:Asn51 (2.10 Å), A:Asn626-B:Ser80 (2.10 Å), A:Tyr811-B:Lys22 (2.20 Å), and A:Ser795-B:Tyr71 (2.30 Å). The HSP90 interface again highlights NSP13 B:Lys22 and B:Asn51 as common contact residues (both seen in HSP40/HSP70 interfaces), and A:Ser795 contributes multiple contacts to NSP13 B:Tyr71 and B:Ser69, suggesting a defined contact patch on HSP90.

The ODC-NSP13 complex (Figure 6) displays the largest number of hydrogen bonds (ten): A:Val103-B:Ser231 (2.00 Å), A:Arg402-B:Ser189 (2.10 Å), A:Asn3-B:Glu168 (2.10 Å), A:Asn3-B:Thr169 (2.10 Å), A:Asn6-B:Glu168 (2.20 Å), A:Asn3-B:Lys171 (2.20 Å), A:Thr404-B:Ser189 (2.30 Å), A:Lys148-B:Glu364 (2.30 Å), A:Asn125-B:Arg389 (2.30 Å), and A:Glu7-B:Thr153 (2.40 Å). ODC also forms a salt bridge via A:Glu106-B:Arg21 (3.30 Å). Of particular note is ODC’s residue Asn3, which forms three distinct hydrogen bonds to NSP13 residues B:Glu168, B:Thr169, and B:Lys171, marking A:Asn3 as an ODC hotspot at the interface. All distances reported here are taken from the BioLuminate v4.6 interaction output and are listed in Supplementary Table S2. As these measurements were derived from static docked conformations, follow-up molecular dynamics validation was carried out to assess persistence and functional consequences of the identified contacts.

### 3.3. Molecular Dynamics Simulations

**Figure 7:**
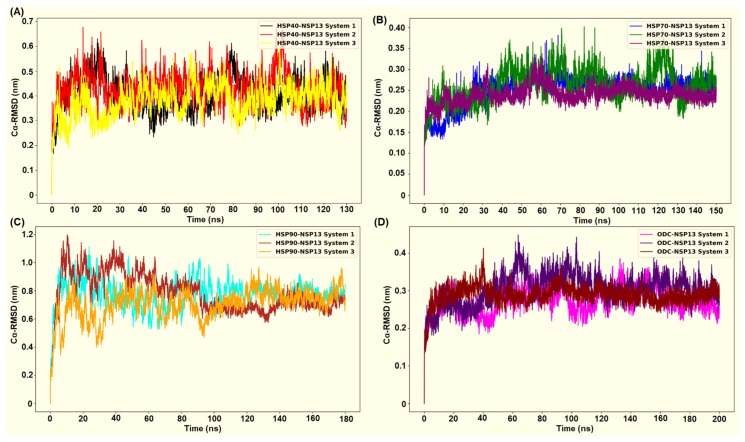
The root mean square deviations and their replicates were generated using *gmx rmsd*: (**A**) HSP40-NSP13 complex; (**B**) HSP70-NSP13 complex; (**C**) HSP90-NSP13 complex; (**D**) ODC-NSP13 complex.

Molecular Dynamics (MD) simulations were performed for 130 ns (HSP40-NSP13 complex), 150 ns (HSP70-NSP13 complex), 180 ns (HSP90-NSP13 complex), and 200 ns (ODC-NSP13 complex). Alpha-carbon (Cα) root mean square deviation (RMSD) plots were used to evaluate the structural stability of NSP13 in complex with four different host proteins. Figure 7A–D show the RMSD plots generated for all four protein complexes in triplicate. The RMSD plots indicate that all four complexes undergo structural adjustment before equilibrating and stabilising adjustment.

After the initial equilibration phase, the HSP70-NSP13 complexes attained the lowest structural deviations of all systems (mean RMSD ± SD: 0.25 ± 0.03 nm; Table S3), thus indicating that they formed a relatively rigid complex. This observed rigidity is conserved across all replicates, with system 3 (purple plot) displaying reduced deviations relative to the other replicates, indicating an even more rigid complex structure (Figure 7B). The ODC-NSP13 complexes were the next most stable, with low RMSD values and tight convergence among replicates (0.29 ± 0.03 nm; Table S3), indicating less structural deviations (Figure 7D). The HSP40-NSP13 complexes displayed moderate RMSD values (0.41 ± 0.06 nm; Table S3), consistent with modest conformational sampling, whilst maintaining their overall structural integrity (Figure 7A). In contrast, HSP90-NSP13 complexes sampled a larger conformational space, displaying higher RMSD values (0.76 ± 0.11 nm; Table S3) (Figure 7C).

**Figure 8:**
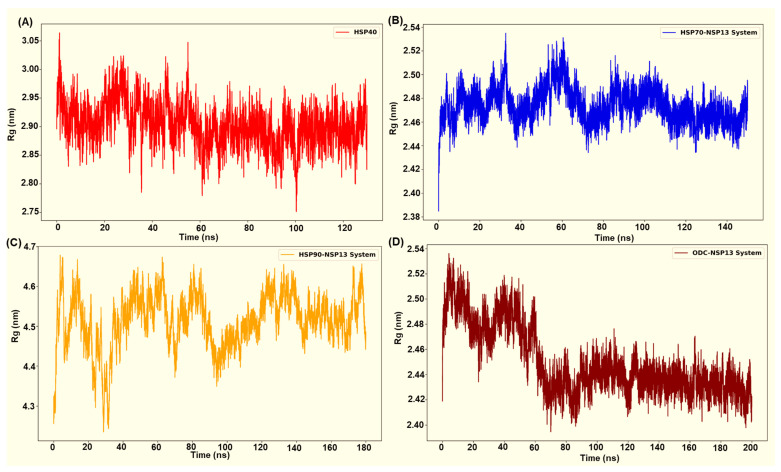
Radius of gyration of complex systems analysed from (**A**) HSP40-NSP13 complex, (**B**) HSP70-NSP13 complex, (**C**) HSP90-NSP13 complex, and (**D**) ODC-NSP13 complex.

The radius-of-gyration (Rg) time plots display the compactness of the protein complexes over time. The computed residue Rg plots (Figure 8A–D) revealed system-specific differences in global compactness that closely aligned with the behaviour of the RMSD for each complex. The selected HSP70-NSP13 system (HSP70-NSP13 system 3) displayed low and tightly distributed Rg values (2.47 ± 0.00 nm), maintaining a compact global structure throughout the trajectory with minor fluctuations indicating structural rearrangements (Figure 8B). This observed compactness mirrors the low RMSD reported for the HSP70-NSP13 complex (0.25 ± 0.01 nm). The ODC complex initially displayed a higher Rg value (from 0 to 60 ns). However, the plot displayed a progressive compaction across the simulation trajectory, settling into a more compact distribution with low Rg values (2.45 ± 0.00 nm). The low RMSD that converges during the trajectory and the decreasing Rg toward a compact value indicate progressive stabilisation of the OD-NSP13 complex (Figure 8D).

Rg for the HSP40-NSP13 complex (Figure 8A) exhibited intermediate compactness. Figure 8A displays fluctuations about a stable mean (2.89 ± 0.00 nm) with no visible deviations from the trend. This corresponds to the modest RMSD values (0.41 ± 0.02 nm), suggesting that the complex retains overall structural integrity with modest conformational sampling, likely due to the flexible linkers. The HSP90-NSP13 complex exhibited markedly high Rg values (4.51 ± 0.00 nm), with large fluctuations, indicating a dynamically flexible and mobile complex. This parallels the RMSD value previously mentioned (0.76 ± 0.04 nm). This suggests that the complex samples multiple, distinct conformational substates that increase the complex’s overall spatial extent.

**Figure 9:**
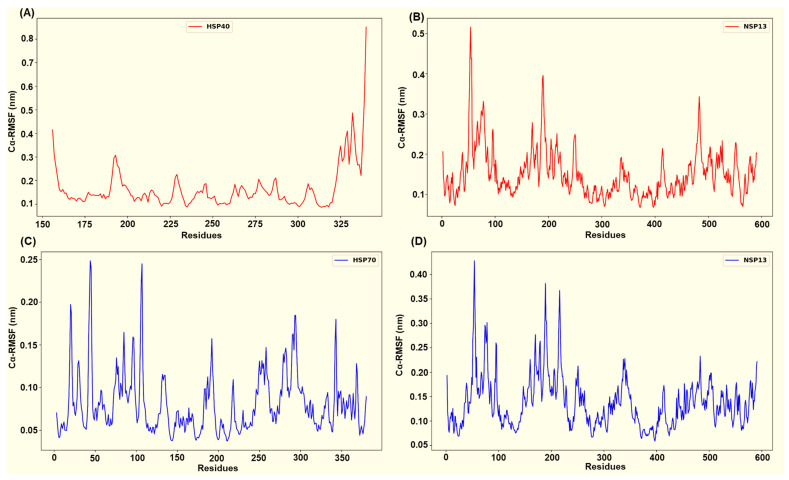
Captured Cα-RMSF plots for (**A**) the host HSP40 protein and (**B**) viral NSP13 protein from the HSP40-NSP13 protein complex, and (**C**) the host HSP70 protein and (**D**) viral NSP13 protein from the HSP70-NSP13 protein complex.

**Figure 10:**
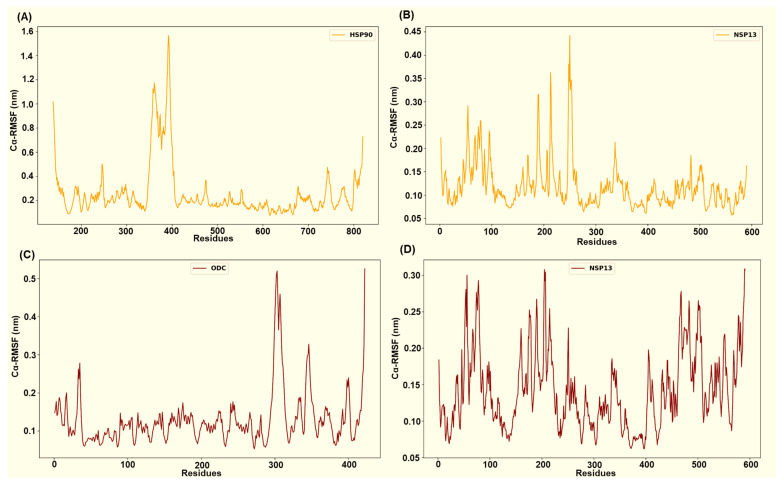
Captured Cα-RMSF plots for (**A**) the host HSP90 protein and (**B**) viral NSP13 protein from the HSP90-NSP13 protein complex, and (**C**) the host ODC protein and (**D**) viral NSP13 protein from the ODC-NSP13 protein complex.

Across the four simulated NSP13–host complexes, per-residue root mean square fluctuation (RMSF) profiles (Figure 9 and Figure 10) highlight distinct flexibility patterns in each complex. In all systems, the residues that participate in persistent interfacial polar contacts tend to display reduced fluctuation relative to the RMSF chain mean. In contrast, non-interacting surface loops retain the largest peaks. The HSP40-NSP13 protein complex displayed moderate chain mobility (HSP40’s mean Cα-RMSF ~ 0.16 ± 0.09 nm; NSP13’s mean Cα-RMSF ~ 0.15 ± 0.06 nm). Docking-identified interface residues from the host protein, HSP40 (Thr205, Glu207, Lys209, Gly215, Lys217, Asp277), displayed low residue flexibility as shown in Figure 9A. On the viral side, the salt-bridge-interacting residue, Lys22, and the Thr188-Thr190 cluster exhibit reduced flexibility while more distal contact residues, such as His230 and Arg338, remain moderately mobile (Figure 3 and Figure 9B).

The host HSP70 from the HSP70-NSP13 complex is markedly rigid (mean Cα-RMSF ~ 0.08 ± 0.04 nm; Figure 9C) while NSP13 retains moderate mobility (mean Cα-RMSF ~ 0.14 ± 0.05 nm; Figure 9D). HSP70 interface residues Glu108, Glu109, Asn111, Asp139, Gln161, Asp228, and Gln327 consistently display Cα-RMSF values well below the HSP70 overall mean. On the other hand, the NSP13 interface residues, notably Asn51, Tyr71, the Thr188-Thr193 region, Tyr217, and Ser234, show moderate stabilisation (slightly reduced RMSF relative to the NSP13 mean) rather than complete immobilisation.

The HSP90-NSP13 system displays the largest host residue mobility (HSP90’s mean Cα-RMSF ~ 0.26 ± 0.22 nm; Figure 10A), while the viral chain remains comparatively restrained (NSP13’s mean Cα-RMSF ~ 0.12 ± 0.05 nm; Figure 10B). The identified HSP90 interface residues (Asn626, Thr716, Thr791, Ser795, Gln804, and Tyr811) displayed residue flexibility, which is consistent with substantial host conformational sampling. In contrast, conserved NSP13 contact residues (Asn51, Tyr71, Lys22, Ser69, His78, and Ser80) exhibit substantially lower Cα-RMSF values, indicating repeated and persistent engagement by polar contacts despite the host’s mobility. The ODC-NSP13 complex demonstrates localised stabilisation that is consistent with a compact bound state (ODC’s mean Cα-RMSF ~ 0.13 ± 0.07 nm; NSP13’s mean Cα-RMSF ~ 0.14 ± 0.05 nm; Figure 10C,D). ODC interface residues (Asn3, Asn6, Glu7, Val103, Glu106, Asn125, Lys148, Arg402, and Thr404) exhibit reduced Cα-RMSF relative to the ODC chain mean. The corresponding NSP13 interface residues (Arg21, Thr153, Glu168, Thr169, Lys171, Ser189, Ser231, Glu364, and Arg389; Figure 6 and Figure 10D) similarly exhibit reduced Cα-RMSF relative to the NSP13 mean and other neighbouring residues.

### 3.4. Cluster Analysis Results

Conformational clustering was employed to elucidate whether each complex samples a single dominant basin or multiple, distinct conformational states. The reported cluster populations, within-cluster RMSD values, and centroids are taken from the clustering output (Tables S4–S7).

GROMOS clustering of the last 30 ns of the HSP40-NSP13 trajectory displayed six clusters (Table S4). The largest cluster (cluster 1) comprises approximately 70% of frames, with a low within-cluster RMSD (0.20 nm). Minor clusters together account for the remaining percentage, including several small substates and rare single-frame outliers. The HSP40-NSP13 trajectory predominantly samples a single conformational basin but occasionally transitions between alternative substates. Thus, the moderate global RMSD (0.41 ± 0.06 nm; Figure 7A) reported for this system primarily reflects inter-cluster differences rather than poor sampling within each basin. Similarly, the HSP40-NSP13 Rg dynamic (Figure 8A) behaviour is consistent with cluster occupancy and transitions.

For HSP70-NSP13, cluster analysis revealed a single dominant conformational basin encompassing all the analysed frames (cluster 1, comprising 151 frames), with a small within-cluster RMSD to the centroid (~0.12 nm), and a centroid occurring late in the trajectory (~134 ns; Table S5). This indicates that the system samples tightly around a single conformational state in the last 30 ns of the simulation. The single-cluster sampling of the HSP70-NSP13 complex correlates with the observed low and tightly distributed radius of gyration (Figure 8B) for this system, thus supporting the interpretation that HSP70 stabilises the viral protein into a compact and rigid assembly.

Clustering analysis of the HSP90-NSP13 trajectory (151 frames) yielded seven clusters, with one dominant cluster (cluster 1, accounting for ~69.5% of the frames), three well-populated clusters (clusters 2–4), and three single-frame outliers (clusters 5–7; Table S6). Within-cluster RMSDs are relatively low (0.18 to 0.20 nm), though the global RMSD is large (0.76 ± 0.11 nm) because the centroids are separated by substantial inter-cluster distances. In short, HSP90-NSP13 samples multiple distinct, well-defined basins. The HSP90-NSP13 Rg plot displayed high mean Rg and large amplitude fluctuations (Figure 8C). Different clusters represent distinct global states of the complex, with some centroids expanding more than others. Cluster switching, particularly between the dominant cluster (cluster 1) and secondary clusters (clusters 2–4), produced the observed significant variability.

The ODC-NSP13 trajectory returned a single conformational basin encompassing all the analysed frames (cluster 1, with 151 frames). The within-cluster RMSD to the centroid was relatively low (0.15 nm), and the centroid occurred at approximately 189.4 ns with a centroid RMSD of 0.13 nm (Table S7). This result is consistent with the late convergence seen in the Rg and RMSD traces (Figure 8D), which supports the interpretation that ODC-NSP13 attains a compact, stable bound state during the simulated window.

### 3.5. Intermolecular Interactions and Binding Affinity of Host Factor–NSP13 Complexes

**Figure 11:**
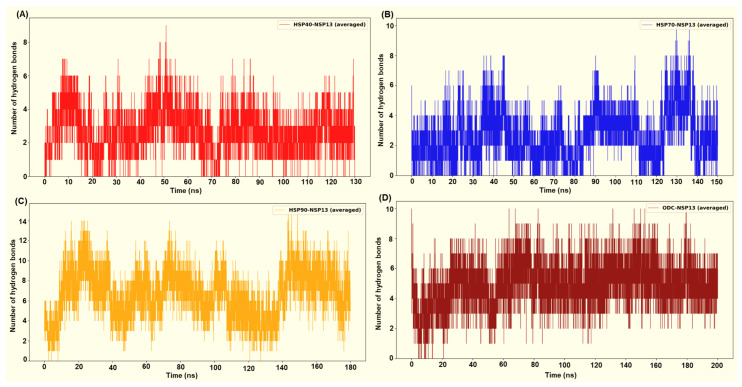
Intermolecular hydrogen bond interactions between the host proteins and the viral protein from (**A**) HSP40-NSP13 systems, (**B**) HSP70-NSP13 systems, (**C**) HSP90-NSP13 systems, and (**D**) ODC-NSP13 systems.

Following the structural and dynamic characterisation, the specific intermolecular interactions and binding affinity for each host factor–NSP13 complex were quantitatively evaluated. Intermolecular hydrogen bond (H-bond) interactions throughout the simulation trajectories were computed to identify key polar contacts crucial for complex stability (Figure 11). Furthermore, the HawkDock server was employed to estimate each complex’s binding free energy (ΔG), providing a thermodynamic measure of the interaction strength (Table 2). Overall, the H-bond interaction plots and the HawkDock/MM-GBSA [28] binding energies consistently correlated across the four host factor–NSP13 complexes. Complexes with higher and more persistent interfacial H-bond counts, generally displayed more favourable (more negative) ΔG. In contrast, systems that exhibited few transient H-bonds displayed weaker ΔG.

HSP90 in complex with NSP13 exhibited the most persistent H-bond interaction count, with peaks up to approximately 12 to 15 H-bonds throughout the simulation (Figure 11A). Complexes displaying higher and more persistent interfacial H-bonds generally display more favourable (more negative) ΔG, while systems with few and transient H-bonds exhibit weaker binding energies. From the MM-GBSA calculations, the HSP90-NSP13 complex exhibited the most favourable binding free energy (ΔG = −82.41 kcal/mol; Table 2). This highly negative ΔG is attributed to the numerous polar contacts previously depicted, substantial hydrophobic packing, and a modest solvent-accessible surface area (SASA; Table 2). The HSP90-NSP13 system’s large RMSD and Rg values, along with multiple clusters, indicate that the complex adopts several distinct global conformational clusters (different centroids). Importantly, these conformational clusters form stabilising contacts that contribute to the high H-bond count and, in turn, the high binding free energy [30].

The ODC-NSP13 system displayed moderate to high H-bond counts, as shown by Figure 11D. These H-bond counts contribute to the second most favourable binding free energy (ΔG = −75.03 kcal/mol; Table 2). The single-cluster occupancy and convergent Rg of the ODC-NSP13 complex indicate that the complex samples one dominant binding mode substantially stabilised by the displayed polar contacts. Compared to HSP90, the binding energy favourability of the ODC-NSP13 complex stems from a stable binding geometry with low conformational heterogeneity. ODC forms a stable, well-packed interface that combines favourable electrostatics and van der Waals (vdW) contacts, while avoiding hefty desolvation penalties (Table 2). The HSP40-NSP13 complex exhibited moderate H-bond interactions, ranging from zero to nine (Figure 11A). Compared to the binding free energies of the HSP90-NSP13 and ODC-NSP13 complexes, the HSP40-NSP13 complex displayed an intermediate binding free energy (ΔG = −42.62 kcal/mol; Table 2). The HSP40-NSP13 system exhibited stabilising polar contacts, fewer than those observed in the HSP90/ODC-NSP13 systems. It also showed a positive electrostatic interaction energy, indicating net repulsive Coulombic interactions (Table 2). The trajectory’s sampling of a central conformational cluster (cluster 1; Table S2) explains why the complex retains an overall structural integrity while permitting small local rearrangements, as shown by minor clusters or spikes.

The HSP70-NSP13 complex was identified as the most conformationally rigid, with low RMSD and Rg values, and a single cluster. The system exhibited moderate and occasionally elevated H-bond counts, with peaks in the range of approximately 8–10 (Figure 11B). This H-bond count is less sustained than those from the HSP90/ODC complexes. The complex exhibited the least binding free energy (ΔG = −35.71 kcal/mol; Table 2) of all four complexes. The low binding free energy suggests that the interaction interface has a limited buried hydrophobic area and that the polar groups at the interaction surface are not optimally oriented.

## 4. Discussion

Utilising molecules such as RNA, DNA, and proteins is crucial for developing pharmaceuticals and the therapeutic sector. Thus, it is essential to understand the roles of polyamines and heat shock proteins in the growth, differentiation, and development of viruses [31]. It is commonly known that for viruses to thrive and spread, they need to compete with the molecules of their host to survive. Therefore, identifying and developing COVID-19 treatments require an understanding of the roles that polyamines and HSPs play in the evolution and growthof viruses [32]. Most species, including viruses like the coronavirus, depend on HSPs as vital components of their cellular systems to maintain proteome balance. Stated differently, HSPs act as guardians of the virus’s cellular structure, enabling it to adapt to the host [33].

Coronavirus entry factors have been reported in numerous studies. When SARS-CoV-2 enters the human host, it utilises the host’s protein production machinery, specifically ribosomes, polyamines, and heat shock proteins, for its replication [34]. Ornithine decarboxylase is an enzyme involved in polyamine synthesis, catalysing the decarboxylation process of ornithine to form putrescine. Polyamines play various roles inside the cellular system due to their polycationic nature [35]. Polyamines promote parasite health and proliferation, while heat shock proteins maintain the parasite’s proteome [36]. In this study, the findings revealed interactions between polyamines and heat shock proteins, with some amino acids being particularly notable for their interactions with polyamines and chaperones when docked with the SARS-CoV-2 helicase (NSP13).

The members of the human heat shock protein 40 (HSP40) family are known as co-chaperone molecules because they serve as the body’s first line of defence when a foreign substance enters. HSP40 contains a J domain that activates HSP70. HSP40 and HSP70 are typically located in the cytosol, making it easier for them to interact with one another during the protein modification process [37]. HSP70 consists of two primary functional domains—the N-terminus nucleotide-binding domain (NBD) and the C-terminus substrate-binding domain (SBD)—both of which play critical roles in HSP70 function. The NBD provides energy by ATP hydrolysis, whereas the SBD binds to substrates and initiates the folding process. The intricate folding mechanism requires strong interaction between the substrate protein and HSP70 [38]. In rare cases, HSP90 steps into the fold for substrates that HSP70 cannot completely fold; hence, it was prudent to investigate the potential interactions between SARS-CoV-2 NSP13 and HSP90. A stable receptor–substrate complex is also necessary, as this protein must be strongly bound to its substrate. As expected, the numerous bonds between these two proteins resulted in a stable complex [39]. ODC is an amino acid dehydrogenase (ADD) that functions on PLP. Based on biochemical studies, ODC is classified as a heterodimer, meaning it has two active sites, each containing residues from both subunits [40]. A pyruvate group is produced at the N-terminus of the chain because of the internal serinolysis reaction that splits the backbone into subunits, the latter of which is the smallest subunit. Drug discovery has extensively utilised bioinformatics, which provides a straightforward platform for developing novel drugs.

All results are derived from docking, molecular dynamics, and comparative MM-GBSA rescoring. These methods generate testable hypotheses about likely interfaces and hotspot residues. Claims regarding biological function or therapeutic targeting are therefore speculative until supported by experiments. Our docking and MD analyses consistently indicate that SARS-CoV-2 NSP13 can potentially bind to both heat shock proteins and the polyamine enzyme ODC, but with different interface characteristics and stabilities. The docking interfaces were dominated by polar interactions (hydrogen bonds and salt bridges), notably in HSP90-NSP13 and ODC-NSP13 complexes (Figure 5, Figure 6 and Figure 11C,D), reflecting the strong electrostatic binding observed in MM-GBSA calculations (Table 2). The negative ΔG for HSP90-NSP13 (−82.41 kcal/mol) and ODC-NSP13 (−75.03 kcal/mol) complexes implies tight binding, whereas HSP40-NSP13 (−42.62 kcal/mol) and HSP70-NSP13 (−35.71 kcal/mol) complexes exhibited weaker affinities. Notably, the electrostatic contributions from the MM-GBSA decomposition were highly favourable for HSP90/ODC systems, but unfavourable for HSP40/70 systems, suggesting the latter rely more on van der Waals interactions. The ranking of these binding energies correlates with the more extensive polar contacts observed in docking for HSP90/ODC (Figure 5 and Figure 6) than in HSP40/70 (Figure 3 and Figure 4).

The MD simulations refined these observations by revealing which of the docked poses persisted. The HSP70-NSP13 and ODC-NSP13 complexes remained remarkably compact (stable RMSD/Rg) and converged onto a single dominant conformation. This suggests that the initial docking poses are potentially close to the true binding mode (further investigation is required), with interface contacts being preserved mainly throughout the trajectory. By contrast, the HSP90-NSP13 complex exhibited larger fluctuations and sampled multiple clusters, indicating a significant rearrangement of the docking interface. The HSP40-NSP13 system displayed intermediate behaviour, forming stable contacts that also reconfigured into a second population. This interpretation is consistent with the general findings that rigid docking overestimates binding complementarity, whereas MD allows relaxation and potentially breaks or forms additional contacts [41].

Analysis of the interaction networks, H-bond persistence, and residue RMSF time series identified common ‘hotspot’ residues on the viral NSP13 protein. Residues Lys22 and Asn51 in the N-terminal zinc-binding domain (ZBD; mapped by residues ~1–100) repeatedly participated in hydrogen bond and salt bridge interactions across several complexes with reduced RMSF in MD, and Tyr71 appeared in HSP40-NSP13 and ODC-NSP13 contacts [42,43]. In contrast, NSP13 residues at positions 188–190 located within the helicase core domains were observed to contribute to the interface interaction in different systems. The residue Gly364 lies in RecA domain 2A, which is involved in ATP hydrolysis. Persistent HSP90-NSP13 contacts involved mainly ZBD residues, whereas ODC engaged more of the helicase core [43]. The overlap of interacting residues among systems implies that these positions are structural hotspots on NSP13, possibly critical for its function and interactions.

Our findings align with known roles of host chaperones and polyamine metabolism in viral replication. Heat shock proteins have been reported to frequently assist viruses by folding nascent viral proteins or stabilising replication complexes [44,45,46]. HSP90 has been reported to be implicated in the folding of polymerases or capsids of many viruses, and its inhibition dampens SARS-CoV-2 replication in culture [44]. HSP40 and HSP70 co-chaperone functions also support viral life cycles [45]. Similarly, viruses often upregulate or co-opt polyamine synthesis for efficient replication [47,48]. ODC is the rate-limiting enzyme in polyamine synthesis, and depletion of polyamines via ODC inhibition (for example, α-Diflouromethylornithine) strongly reduces coronavirus attachment and replication [48]. The predicted strong binding of NSP13 to ODC therefore suggests a mechanism by which SARS-CoV-2 potentially recruits polyamine metabolism. By physically interacting with ODC, NSP13 could potentially localise to sites of polyamine production or stabilise ODC, thereby ensuring high polyamine levels for RNA replication. While to the best of our knowledge, no previous study has directly reported ODC-NSP13 binding, our findings extend the paradigm that viral helicases may engage host metabolic enzymes to facilitate genome processing [47,48].

## 5. Conclusions

Our computational analyses predict that SARS-CoV-2 NSP13 is capable of forming distinct interfaces with host heat shock proteins and ODC, and identify candidate NSP13 residues that may act as interaction hotspots. The interactions between NSP13 and HSP families (HSP40, HSP70, and HSP90), as well as ODC, revealed varying levels of binding stability and energy, emphasising their diverse contributions to viral protein folding and replication. Our in-silico analysis suggests that SARS-CoV-2 NSP13 can form stable complexes with host HSP70 and ODC with specific interface residues, whereas its association with HSP90 appears strong but more transient. These findings are consistent with the known roles of host chaperones and polyamine metabolism in viral replication. We propose that targeting NSP13’s recruitment of HSP/ODC could disrupt viral replication. Overall, these findings provide us with deeper mechanistic insights into virus–host protein interactions, thus supporting their potential as therapeutic targets in COVID-19 drug discovery.

### Future Work

To translate our computational hypotheses into mechanistic insight and tractable intervention strategies, we must first convert contact-based signals into quantitative energetic evidence. The highest-priority task is per-residue energetic decomposition and in silico alanine scanning of the identified interface residues to further identify experimentally testable hotspots and enable targeted mutagenesis to validate the computational interface. Building on the validated hotspots, future work will also include pharmacophore development and virtual screening of small-molecule compounds capable of exploiting the most stable interaction features of the host–NSP13 complexes and inhibiting/disrupting the interaction [49,50,51]. Ultimately, these hypotheses require experimental testing to confirm the biological significance of host–NSP13 interactions. Quantitative biophysical assays (for example, surface plasmon resonance) and mutational analyses will be performed to explore the predicted contributions of hotspot residues. Together, these steps will refine the mechanistic model proposed here and lay the groundwork for targeted intervention strategies against NSP13-mediated host factor recruitment.

## Figures and Tables

**Table 1:** Selected templates for homology modelling of host and SARS-CoV-2 NSP13 proteins.

Target Protein (Accession)	Template PDB ID (Chain)	Organism	Sequence Identity (%)	E-Value
HSP40	3AGZ; (A)	*Homo sapiens*	100	2.68 × 10^−134^
HSP70	6GFA; (A)	*Homo sapiens*	75	0
HSP90	7KRJ; (A)	*Homo sapiens*	100	0
ODC	2OO0; (A)	*Homo sapiens*	100	0
NSP13	6SZL	*SARS-CoV-2*	100	0

**Table 2:** MM-GBSA binding free energy decomposition of host–NSP13 complexes computed from HawkDock server (v2).

Complex	Van der Waals Interaction Energy Contribution (kcal/mol)	Electrostatic Interaction Energy Contribution (kcal/mol)	Generalised Born Solvation Energy Contribution (kcal/mol)	Solvent-Accessible Surface Area Contribution to the Energy (kcal/mol)	Total ΔG (kcal/mol)
HSP90-NSP13	–151.42	–615.11	702.57	–18.46	–82.41
ODC-NSP13	–122.57	–552.32	64.61	–14.74	–75.03
HSP40-NSP13	–84.56	107.12	–54.50	–10.68	–42.62
HSP70-NSP13	–98.22	127.35	–52.67	–12.17	–35.71

## Data Availability

The original contributions presented in this study are included in the article/Supplementary Materials. Further inquiries can be directed to the corresponding author.

## Supplementary Materials

The following supporting information can be downloaded at https://www.mdpi.com/article/10.3390/cimb48010080/s1.
