# Supplementary material for: SARS-CoV-2 Helicase (NSP13) Interacts with Mammalian Polyamine and HSP Partners in Promoting Viral Replication
Source: Curr Issues Mol Biol. 2026 Jan 13;48(1):80. doi: 10.3390/cimb48010080 (PMC12840268; doi:10.3390/cimb48010080)
Supplement: Supplementary file 1 [file cimb-48-00080-s001.zip › cimb-3928735-supplementary.pdf]

current issues in molecular biology

Article

SARS-Cov-2 Helicase (NSP13) Interacts with Mammalian Polyamine and HSP Partners in Promoting Viral Replication

Zingisa Sitobo [1], Liberty T Navhaya [2], Nqumla Ntombekhaya [1], Masenya Madipoane [4], Matsheliso Molapo [4], Yamkela Mthembu [3], Godlo Sesethu [4] and Xolani H Makhoba [4]*

1 Department of Biochemistry and Microbiology, University of Fort Hare, Alice Campus, Alice 5700, South Africa
2 Department of Biochemistry, Microbiology and Biotechnology, University of Limpopo, Turfloop Campus, Sovenga 0727, South Africa
3 Department of Life and Consumer Sciences, College of Agriculture and Environmental Sciences, University of South Africa (UNISA), Florida Campus, Roodepoort 1709, South Africa
4 Human Genetics Department, Computational Biology Division, Faculty of Health Sciences, University of Cape Town, Barnard Fuller Building, Anzio Rd, Observatory, Cape Town 7935, South Africa.

* Correspondence: makhoxh@unisa.ac.za

current issues in molecular biology

MDPI

Table S1. Sequence analysis of selected host proteins (HSP40, HSP70, HSP90, and ODC enzyme) and SARS-CoV-2 helicase.

Protein name	Organism	Accession No.	Assembly	Chromosome	Locus	Gene ID	Exons	Introns
Heat shock proteins								
Heat shock protein 40 (HSP40)	Human	P25685.4	GRCh38.p14	19	NC_000019.10	3337	8	7
Heat shock protein 70 (HSP70)	Human	NP_002145.3	GRCh38.p14	5	NC_000005.10	3308	19	18
Heat shock protein 90 (HSP90)	Human	NP_001017963.2	GRCh38.p14	14	NC_000014.9	3320	13	12
Polyamine								
Ornithine decarboxylase	Human	P11926.2	GRCh38.p14	2	NC_000002.12	4953	13	12
Virus								
SARS-CoV-2 helicase (NSP13)	SARS-CoV-2	YP_459942.1	-	-	-	3200429	-	-

current issues in
molecular biology

MDPI

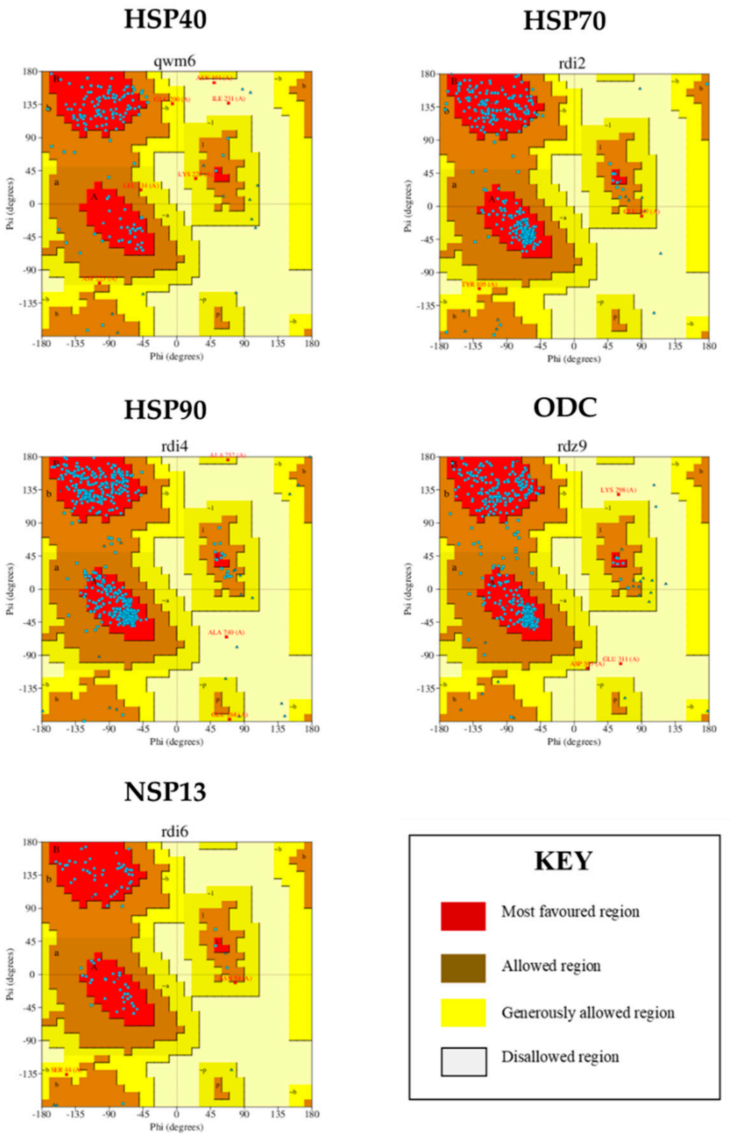

Figure S1. Ramachandran plots of the 3D model structures for HSP40, HSP70, HSP90, ODC enzyme, and SARS-CoV-2 helicase (NSP13) generated using PDBsum.

current issues in molecular biology

MDPI

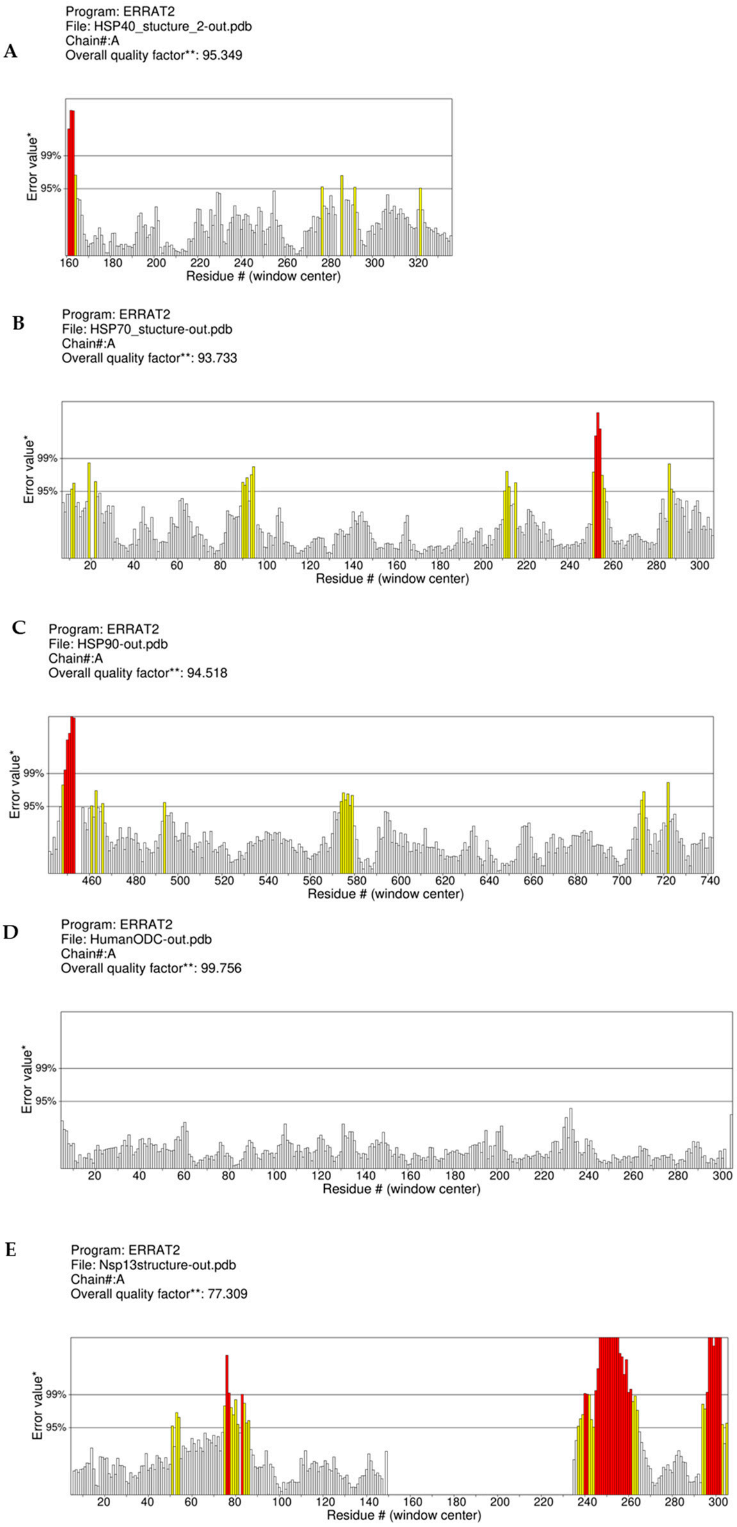

Figure S2. Results of the validation analysis for protein crystal structures of **(A)** HSP40, **(B)** HSP70, **(C)** HSP90, **(D)** ODC enzyme, and **(E)** SARS-CoV-2 helicase (NSP13) were obtained using ERRAT.

current issues in molecular biology

MDPI

Table S2. Summary of protein–protein contacts between host proteins (molecular chaperones and polyamine; chain A) and SARS-CoV-2 NSP13 (chain B) identified by BioLuminate v4.6.

Protein Complex	Host Protein Residues	Viral Protein Residues	Distance (Å)	Specific Interactions
HSP40-NSP13	A:Gly 215	B:Thr 190	2.00	1x hb to B:Thr 190
	A:Lys 217	B:Thr 190	2.10	1x hb to B:Thr 190
	A:Lys 217	B:Thr 188	2.10	1x hb to B:Thr 188
	A:Lys 209	B:Tyr 185	2.30	1x hb to B:Tyr 185
	A:Glu 207	B:Hie 230	2.30	1x hb to B:Hie 230
	A:Thr 205	B:Arg 338	2.40	1x hb to B:Arg 338
	A:Asp 277	B:Lys 22	3.30	1x salt bridge to B:Lys 22
HSP70-NSP13	A:Gln 327	B:Thr 193	1.80	1x hb to B:Thr 193
	A:Asp 228	B:Thr 190	1.90	1x hb to B:Thr 190
	A:Asn 111	B:Asn 51	1.90	1x hb to B:Asn 51
	A:Asp 139	B:Glh 364	2.00	1x hb to B:Glh 364
	A:Glh 109	B:Asn 51	2.00	1x hb to B:Asn 51
	A:Gln 161	B:Ser 234	2.10	1x hb to B:Ser 234
	A:Gln 327	B:Tyr 217	2.20	1x hb to B:Tyr 217
	A:Glu 108	B:Tyr 71	2.40	1x hb to B:Tyr 71
HSP90-NSP13	A:Thr 716	B:His 78	1.90	1x hb to B:His 78
	A:Ser 795	B:Ser 69	2.00	1x hb to B:Ser 69
	A:Gln 804	B:Lys 22	2.10	1x hb to B:Lys 22
	A:Thr 791	B:Asn 51	2.10	1x hb to B:Asn 51
	A:Asn 626	B:Ser 80	2.10	1x hb to B:Ser 80
	A:Tyr 811	B:Lys 22	2.20	1x hb to B:Lys 22
	A:Ser 795	B:Tyr 71	2.30	1x hb to B:Tyr 71
ODC-NSP13	A:Val 103	B:Ser 231	2.00	1x hb to B:Ser 231
	A:Arg 402	B:Ser 189	2.10	1x hb to B:Ser 189
	A:Asn 3	B:Glu 168	2.10	1x hb to B:Glu 168
	A:Asn 3	B:Thr 169	2.10	1x hb to B:Thr 169
	A:Asn 6	B:Glu 168	2.20	1x hb to B:Glu 168
	A:Asn 3	B:Lys 171	2.20	1x hb to B:Lys 171
	A:Thr 404	B:Ser 189	2.30	1x hb to B:Ser 189
	A:Lys 148	B:Glu 364	2.30	1x hb to B:Glu 364
	A:Asn 125	B:Arg 389	2.30	1x hb to B:Arg 389
	A:Glu 7	B:Thr 153	2.40	1x hb to B:Thr 153
	A:Glu 106	B:Arg 21	3.30	1x salt bridge to B:Arg 21

Table S3: Root mean square deviation (RMSD) for three replicate MD simulations of host protein–NSP13 complexes,

Protein Complex	System	Number of frames	Mean RMSD (nm)	Standard Deviation RMSD (nm)	Overall Mean ± SD RMSD (nm)
HSP40–NSP13	HSP40–NSP13 System 1	13001	0.41	0.06	0.41 ± 0.06
	HSP40–NSP13 System 2	13001	0.43	0.06	
	HSP40–NSP13 System 3	13001	0.38	0.06	
HSP70–NSP13	HSP70–NSP13 System 1	15001	0.25	0.03	0.25 ± 0.03
	HSP70–NSP13 System 2	15001	0.26	0.04	
	HSP70–NSP13 System 3	15001	0.24	0.02	
HSP90–NSP13	HSP90–NSP13 System 1	18001	0.78	0.10	0.76 ± 0.11
	HSP90–NSP13 System 2	18001	0.79	0.12	
	HSP90–NSP13 System 3	18001	0.71	0.11	
ODC–NSP13	ODC–NSP13 System 1	20001	0.27	0.03	0.29 ± 0.03
	ODC–NSP13 System 2	20001	0.30	0.04	
	ODC–NSP13 System 3	20001	0.29	0.02	

Table S4. The number of structures in each cluster, the average RMSD, the representative (middle) structure with its corresponding time and RMSD, and the timeframes for each cluster for the HSP40-NSP13 protein complex.

Cluster	Number of Structures	RMSD (nm)	Middle Structure (time, RMSD)	Cluster Members
1	106	0.20	115.6, 0.17	101.2, 102, 102.4, 102.8, 103, 103.4, 103.8, 104, 104.2, 105.4, 105.6, 105.8, 106, 106.2, 106.4, 106.6, 106.8, 107, 107.2, 107.4, 107.6, 107.8, 108, 108.2, 108.4, 108.6, 109, 109.2, 109.4, 109.6, 109.8, 110, 110.2, 111, 111.2, 111.4, 111.6, 111.8, 112, 112.2, 112.4, 112.6, 112.8, 113, 113.2, 113.4, 113.6, 113.8, 114, 114.2, 114.4, 114.6, 114.8, 115.6, 115.8, 116, 116.2, 116.4, 116.6, 116.8, 117, 117.2, 117.4, 117.6, 117.8, 118, 118.2, 118.4, 118.6, 118.8, 119, 119.2, 119.4, 119.6, 119.8, 120, 120.2, 120.4, 120.6, 120.8, 121, 121.4, 121.6, 121.8, 122, 122.2, 122.4, 123.4, 123.6, 123.8, 124, 124.2, 124.4, 124.6, 124.8, 125.4, 127, 127.4, 127.6, 127.8, 128, 129, 129.4, 129.6, 129.8, 130
2	25	0.20	101.6, 0.18	100, 100.2, 100.8, 101.4, 101.6, 102.2, 102.6, 103.2, 103.6, 104.4, 104.6, 104.8, 105, 105.2, 108.8, 110.4, 110.6, 110.8, 115, 115.2, 115.4, 122.8, 123, 128.6, 128.8
3	11	0.21	126.6, 0.18	122.6, 125.2, 125.6, 125.8, 126, 126.2, 126.4, 126.6, 126.8, 127.2, 128.4
4	7	0.19	100.6, 0.17	100.4, 100.6, 101, 101.8, 121.2, 123.2, 125
5	1	—	129.2	129.2
6	1	—	128.2	129.2

Table S5. The number of structures in each cluster, the average RMSD, the representative (middle) structure with its corresponding time and RMSD, and the timeframes for each cluster for the HSP70-NSP13 protein complex.

Cluster	Number of Structures	RMSD (nm)	Middle Structure (time, RMSD)	Cluster Members
1	151	0.12	134, 0.11	120, 120.2, 120.4, 120.6, 120.8, 121, 121.2, 121.4, 121.6, 121.8, 122, 122.2, 122.4, 122.6, 122.8, 123, 123.2, 123.4, 123.6, 123.8, 124, 124.2, 124.4, 124.6, 124.8, 125, 125.2, 125.4, 125.6, 125.8, 126, 126.2, 126.4, 126.6, 126.8, 127, 127.2, 127.4, 127.6, 127.8, 128, 128.2, 128.4, 128.6, 128.8, 129, 129.2, 129.4, 129.6, 129.8, 130, 130.2, 130.4, 130.6, 130.8, 131, 131.2, 131.4, 131.6, 131.8, 132, 132.2, 132.4, 132.6, 132.8, 133, 133.2, 133.4, 133.6, 133.8, 134, 134.2, 134.4, 134.6, 134.8, 135, 135.2, 135.4, 135.6, 135.8, 136, 136.2, 136.4, 136.6, 136.8, 137, 137.2, 137.4, 137.6, 137.8, 138, 138.2, 138.4, 138.6, 138.8, 139, 139.2, 139.4, 139.6, 139.8, 140, 140.2, 140.4, 140.6, 140.8, 141, 141.2, 141.4, 141.6, 141.8, 142, 142.2, 142.4, 142.6, 142.8, 143, 143.2, 143.4, 143.6, 143.8, 144, 144.2, 144.4, 144.6, 144.8, 145, 145.2, 145.4, 145.6, 145.8, 146, 146.2, 146.4, 146.6, 146.8, 147, 147.2, 147.4, 147.6, 147.8, 148, 148.2, 148.4, 148.6, 148.8, 149, 149.2, 149.4, 149.6, 149.8, 150

Table S6. The number of structures in each cluster, the average RMSD, the representative (middle) structure with its corresponding time and RMSD, and the timeframes for each cluster for the HSP90-NSP13 protein complex.

Cluster	Number of Structures	RMSD (nm)	Middle Structure (time, RMSD)	Cluster Members
1	105	0.19	151.8, 0.16	150, 150.2, 150.4, 150.6, 150.8, 151, 151.2, 151.4, 151.6, 151.8, 152, 152.2, 152.4, 152.6, 152.8, 153, 153.2, 153.4, 153.6, 153.8, 154, 154.2, 154.4, 154.6, 154.8, 155, 155.2, 155.4, 155.6, 155.8, 156, 156.2, 156.4, 156.6, 156.8, 157, 157.2, 157.4, 157.6, 157.8, 158, 158.2, 158.4, 158.6, 158.8, 159, 159.2, 159.4, 159.6, 159.8, 160, 160.2, 160.4, 160.6, 160.8, 161, 161.2, 161.4, 161.6, 161.8, 162, 162.2, 162.4, 162.6, 162.8, 163, 163.2, 163.4, 163.6, 163.8, 164, 164.2, 164.4, 164.6, 164.8, 165, 165.2, 165.4, 165.6, 165.8, 166.2, 166.4, 166.8, 167, 167.2, 167.4, 167.6, 167.8, 168.2, 168.4, 168.6, 169, 169.4, 170, 170.4, 171.4, 171.6, 171.8, 173.6, 173.8, 174, 174.2, 175, 175.2, 179.6
2	24	0.19	177, 0.16	172, 172.4, 172.6, 172.8, 173.2, 173.4, 175.4, 175.6, 175.8, 176.2, 176.4, 176.6, 176.8, 177.2, 177.2, 177.4, 177.6, 177.8, 178, 178.2, 178.4, 178.6, 178.6, 179
3	10	0.18	170.2, 0.17	166.6, 168, 168.8, 169.2, 169.6, 169.8, 170.2, 170.6, 170.8, 171
4	9	0.20	179.2, 0.17	171.2, 172.2, 174.4, 174.6, 174.8, 178.8, 179.2, 179.4, 180
5	1	—	166	166
6	1	—	179.8	179.8
7	1	—	173	173

current issues in molecular biology

Table S7. The number of structures in each cluster, the average RMSD, the representative (middle) structure with its corresponding time and RMSD, and the timeframes for each cluster for the ODC-NSP13 protein complex.

Cluster	Number of Structures	RMSD (nm)	Middle Structure (time, RMSD)	Cluster Members
1	151	0.15	189.4, 0.13	170, 170.2, 170.4, 170.6, 170.8, 171, 171.2, 171.4, 171.6, 171.8, 172, 172.2, 172.4, 172.6, 172.8, 173, 173.2, 173.4, 173.6, 173.8, 174, 174.2, 174.4, 174.6, 174.8, 175, 175.2, 175.4, 175.6, 175.8, 176, 176.2, 176.4, 176.6, 176.8, 177, 177.2, 177.4, 177.6, 177.8, 178, 178.2, 178.4, 178.6, 178.8, 179, 179.2, 179.4, 179.6, 179.8, 180, 180.2, 180.4, 180.6, 180.8, 181, 181.2, 181.4, 181.6, 181.8, 182, 182.2, 182.4, 182.6, 182.8, 183, 183.2, 183.4, 183.6, 183.8, 184, 184.2, 184.4, 184.6, 184.8, 185, 185.2, 185.4, 185.6, 185.8, 186, 186.2, 186.4, 186.6, 186.8, 187, 187.2, 187.4, 187.6, 187.8, 188, 188.2, 188.4, 188.6, 188.8, 189, 189.2, 189.4, 189.6, 189.8, 190, 190.2, 190.4, 190.6, 190.8, 191, 191.2, 191.4, 191.6, 191.8, 192, 192.2, 192.4, 192.6, 192.8, 193, 193.2, 193.4, 193.6, 193.8, 194, 194.2, 194.4, 194.6, 194.8, 195, 195.2, 195.4, 195.6, 195.8, 196, 196.2, 196.4, 196.6, 196.8, 197, 197.2, 197.4, 197.6, 197.8, 198, 198.2, 198.4, 198.6, 198.8, 199, 199.2, 199.4, 199.6, 199.8, 200
